# Cumulative Doses to Brain and Other Critical Structures After Multisession Gamma Knife Stereotactic Radiosurgery for Treatment of Multiple Metastatic Tumors

**DOI:** 10.3389/fonc.2018.00065

**Published:** 2018-03-13

**Authors:** Jianling Yuan, Richard Lee, Kathryn Ellen Dusenbery, Chung K. Lee, Damien C. Mathew, Paul Wayne Sperduto, Yoichi Watanabe

**Affiliations:** ^1^Department of Radiation Oncology, University of Minnesota Medical School, University of Minnesota, Minneapolis, MN, United States; ^2^University of Minnesota Medical Center-Fairview Gamma Knife Center, University of Minnesota, Minneapolis, MN, United States

**Keywords:** stereotactic radiosurgery, multisession, brain metastasis, cumulative dose, gamma knife

## Abstract

**Purpose:**

Repeat stereotactic radiosurgery (SRS) is an attractive alternative to whole brain radiation therapy (WBRT) for treatment of recurrent brain metastases (BM). The purpose of this study is to determine the cumulative doses to the brain and critical normal structures in patients who underwent repeat courses of Gamma Knife (GK) SRS.

**Materials and methods:**

We retrospectively identified ten patients who received at least three GK-SRS sessions for multiply recurrent BM at our institution from 2013 to 2016. We used Velocity™ 3.1.0 software to co-register the magnetic resonance imaging images and the dose data of all treatment sessions for each patient. The cumulative doses to brain, lenses, eyes, brainstem, optic nerves, chiasm, and hippocampi were calculated. Dose–volume histograms, as well as the mean, median and maximum doses of these structures, were analyzed.

**Results:**

The median number of SRS was five sessions (range = 3–7 sessions) per patient over a median treatment span of 510 days (112–1,197 days), whereas the median number of metastatic tumors treated per patient was 25.0 (10–63). The median of the total tumor volume was 9.5 cc (2.3–75.9 cc). The median of the mean cumulative dose to the whole brain was 4.1 Gy (1.7–16.4 Gy). The medians of the maximum doses to the critical structures were as follows: brainstem, 6.1 Gy (2.2–28.9 Gy), chiasm, 3.9 Gy (1.8–10.8 Gy), right optic nerve, 2.9 Gy (1.2–9.0 Gy), and left optic nerve, 2.6 Gy (1.0–6.5 Gy). The medians of the mean and maximum cumulative doses to the hippocampi were 3.4 Gy (1.0–14.4 Gy) and 13.8 Gy (1.5–39.3 Gy), respectively. The median survival for the entire cohort was 26.7 months, and no patients developed radiation necrosis.

**Conclusion:**

Our study demonstrated that multisession GKSRS could be delivered with low cumulative doses to critical normal structures. Further studies are required to fully establish its role as an alternative treatment strategy to WBRT for the treatment of multiply recurrent BM.

## Introduction

Brain metastases (BM) are the most common type of intracranial tumors. Older population-based studies in the 1970s and 1980s have shown an incidence rate of up to 10% among patients diagnosed with cancer (1). In more recent studies, the incidence of BM is reported to be closer to 30% (2–4). This was thought to be due to the increased detectability through the use of brain magnetic resonance imaging (MRI) as well as improvements in systemic therapy leading to longer survival. Historically, the median survival after diagnosis with BM was approximately 1 month without any treatment, which may be doubled with the use of steroids (5, 6). Currently, whole brain radiation therapy (WBRT) is the standard treatment option for BM, either alone or in combination with stereotactic radiosurgery (SRS). This is driven by the rationale that the entire brain may be “seeded” with micrometastatic disease since the most common route of dissemination is hematogenous (7). Indeed, multiple retrospective and prospective studies have shown that SRS alone is associated with a higher risk of distant intracranial progression despite its excellent local control (2, 8–11). Therefore, WBRT is often recommended upfront, or after new BMs are found following an initial SRS treatment. However, none of the prospective studies have shown a survival advantage of upfront WBRT plus SRS compared to SRS alone (2, 12, 13). Furthermore, WBRT is well documented to have a negative impact on neurocognitive function, cerebellar function, and quality of life (QOL) (2, 3, 14). This issue is becoming increasingly relevant in the era of targeted therapy and immunotherapy, which has significantly prolonged survival in select patients.

To limit CNS morbidity associated with WBRT, several approaches have been tested, including the concurrent use of memantine (15) and hippocampal sparing (13). Results from radiation therapy oncology group (RTOG) 0933 phase II clinical trial demonstrated that conformal avoidance of the hippocampus during WBRT was associated with preservation of memory and QOL compared with historical series (13). Hippocampal sparing is generally more easily achievable with SRS because of the sharp dose gradient surrounding a target, making repeat courses of SRS an attractive strategy to preserve cognitive function among patients with multiply recurrent BM. While many physicians have become comfortable treating multiple lesions in a single SRS course, repeat SRS is only a recent practice trend. The dosimetric consequence and the clinical outcome of multisession SRS are not well established. We set out to determine the cumulative doses to the brain and critical normal structures in patients who underwent multiple sessions of gamma knife (GK) SRS at our institution.

## Materials and Methods

### Patients

From a prospectively maintained database of patients treated with GKSRS at our institution, we identified all patients who received multisession treatments with a total of at least 10 lesions since 2013. We defined multisession as three or more separate GK courses, each at least 1 month apart. These selection criteria allowed us to analyze patients who had treatments to a large number of lesions over an extended follow-up period. At our institution, patients who have been treated with GKSRS are routinely followed with MRI at 3-month intervals. When new lesions are identified, the decision to offer a repeat course of GKSRS versus WBRT in these patients is jointly made by the treating radiation oncologist and the neurosurgeon, often in the multidisciplinary tumor board setting, after carefully reviewing the MRI imaging, previous treatment records, and systemic disease status. In general, patients with good performance status and well-controlled systemic disease who present with a limited number of new brain lesions deemed to be treatable in one GK session are counseled on the option of repeat SRS. Patients with any type of primary histology were included. Previous treatment with WBRT prior to first GKSRS was allowed. Demographic and tumor-related information was extracted from the electronic medical record. This study was approved by the institutional review board (IRB code number: 0801M23942).

### Radiation Treatment

GKSRS was performed using the Leksell Gamma Knife Model 4 C (Elekta AB, Stockholm, Sweden). All patients were treated with frame-based immobilization and MRI-based treatment planning. Radiation dose was selected primarily based on tumor size according to the RTOG 90-05 trial, with modifications made by the prescribing physician (16). In general, tumors measuring <2, 2–3, and 3–4 cm received 24, 18, and 15 Gy, respectively. Doses were generally prescribed to the 50% isodose line to the Gadolinium-enhanced tumor volume on T1-weighted MRI image without an additional margin. All tumors were treated with a single-fraction SRS.

### Follow-up Imaging

Following each GKSRS, patients are typically followed with repeat brain MRIs at 3-month intervals; sooner if new neurologic symptom develops.

### Dosimetric Analysis

**Figure 1:**
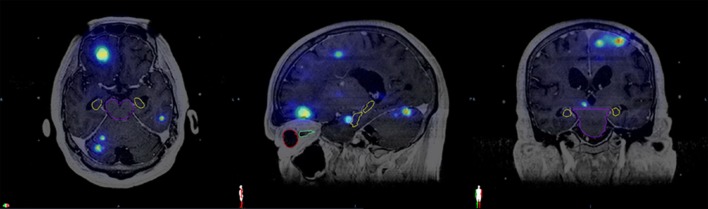
Fused magnetic resonance imaging from the same patient with cumulative isodose distribution displayed in the axial, sagittal, and coronal views along with contours of various organs at risk. Brainstem (purple), hippocampus (yellow), right eye (red), right optic nerve (green).

The dosimetric data and T1-weighted Gadolinium-enhanced MRI images in DICOM format were exported from the GammaPlan^®^ version 10.1.1 to Velocity™ 3.1.0 (Varian Medical Systems, Palo Alto, CA, USA) for each patient. The MRI images and the dose data of all previous sessions were co-registered with the reference data set of the most recent SRS using the rigid-registration technique of Velocity™ (Figure 1). All of the normal structures were contoured on the reference MRI. The hippocampi were contoured according to the RTOG 0933 hippocampal atlas. The cumulative doses for brain, lenses, eyes, brainstem, optic nerves, chiasm, and hippocampi from all GK sessions were calculated by adding the doses at matched spatial locations on the co-registered images using the Velocity™ software. Dose–volume histograms of these structures were generated based on the cumulative doses from all sessions. The median and maximum doses to each structure were recorded.

## Results

### Patient and Treatment Characteristics

We identified a total of 10 patients who underwent three or more GKSRS sessions to treat ≥10 lesions at our institution from 2013 to 2016 (Table 1). Primary diagnoses included melanoma (*n* = 6), small cell lung cancer (*n* = 1), non-small cell lung cancer (*n* = 1), breast cancer (*n* = 1), and renal cell carcinoma (*n* = 1). Six patients were males; four were females, aged between 44 and 70 years old at the time of their first GKSRS treatment. Three patients had extracranial systemic disease at the time of their GKSRS, while the remaining seven patients only had CNS disease. All patients had Karnofsky Performance Status score of 90 or above, and Graded Prognostic Assessment (GPA) ranged between 2 and 3. Information regarding the use of systemic therapy immediately before or after the first GKSRS was also recorded when available. Note that only two patients had prior WBRT.

**Table 1:** Patient characteristics at the first GKSRS.

Pt ID	Age at first GKS (yo)	Gender	WBRT	Primary histology	KPS (%)	Extracranial disease	Peri-GK systemic therapy	GPA
1	61	F	N	Melanoma	90	N	None	3
2	44	F	N	Melanoma	90	N	Interferon in 2010	2.5
3	53	M	Y	SCLC	90	Y	Temodar	2.5
4	46	M	N	NSCLC	100	Y	None	2.5
5	67	M	Y	Melanoma	90	Y	Ipilimumab, pembrolizumab	2
6	70	M	N	Melanoma	90	N	None	2
7	70	M	N	Melanoma	90	N	Interferon, ipilimumab	2
8	49	F	N	Melanoma	90	N	None	2.5
9	44	F	N	Breast	90	N	FEC, Herceptin, Taxol, Abraxane, Tamoxifen in 2012	2
10	59	M	N	RCC	100	N	High-dose IL-2 in 2012	3

*F, female; M, male; WBRT, whole brain radiation therapy; Y, yes; N, no; SCLC, small cell lung cancer; NSCLC, non-small cell lung cancer; RCC, renal cell carcinoma; KPS, Karnofsky Performance Status; GPA, Graded Prognostic Assessment, FEC, 5-FU + epirubicin + cyclophosphamide*.

**Figure 2:**
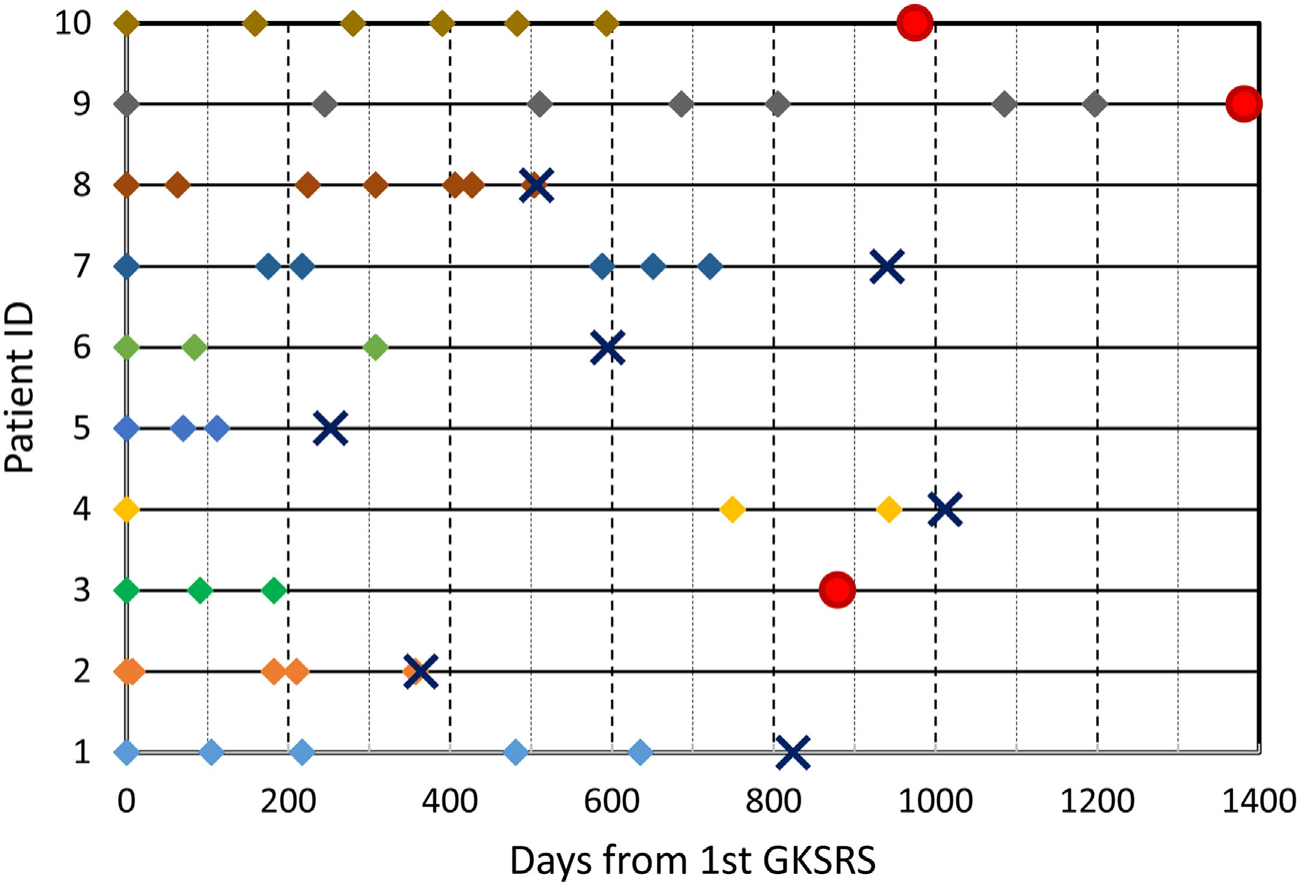
GKSRS treatment timeline and vital status of ten patients. The diamond symbols indicate the time of treatment visit in days since the first GKSRS. The circle indicates last follow up for alive patients, and the cross “X” indicates the time of death.

The median number of SRS sessions was 5 (range, 3–7) per patient. These treatments were delivered over a median treatment span of 510 days (range, 112–1,197 days). Figure 2 depicts the timeline of GKSRS treatment visits for each patient. The total number of metastatic tumors treated per patient ranged from 10 to 63 with a median of 25.0, and the total tumor volume ranged from 2.3 to 75.9 cc (median = 9.5 cc) (Table 2). The median interval to retreatment was 111 days (range, 7–371 days). It should be noted that nearly all of the treatments were directed at new lesions. One patient (Patient #8) had four tumors treated twice, and one tumor treated three times. Patients #1 and #9 each had one tumor treated twice.

**Table 2:** GKSRS treatment characteristics.

Pt ID	Txt span (days)	No. sessions	Total No. mets	Total tumor Vol. (cc)	Brain Vol. (cc)
1	635	5	26	11.16	1,326.40
2	357	5	53	31.94	1,282.80
3	182	3	26	2.29	1,802.10
4	943	3	10	7.90	1,739.40
5	112	3	28	7.61	1,484.60
6	308	3	12	2.26	1,613.80
7	721	6	20	25.26	1,471.40
8	427	7	63	75.94	1,401.00
9	1,197	7	18	26.14	1,369.20
10	593	6	24	7.62	1,288.52
Mean	548	4.80	28.00	19.81	1,477.92
SD	341	1.69	17.06	22.35	184.59
Median	510	5.00	25.00	9.53	1,436.20

*Txt, treatment; Mets, Metastases; Vol, volume, No, number*.

### Cumulative Doses to Critical Structures

**Figure 3:**
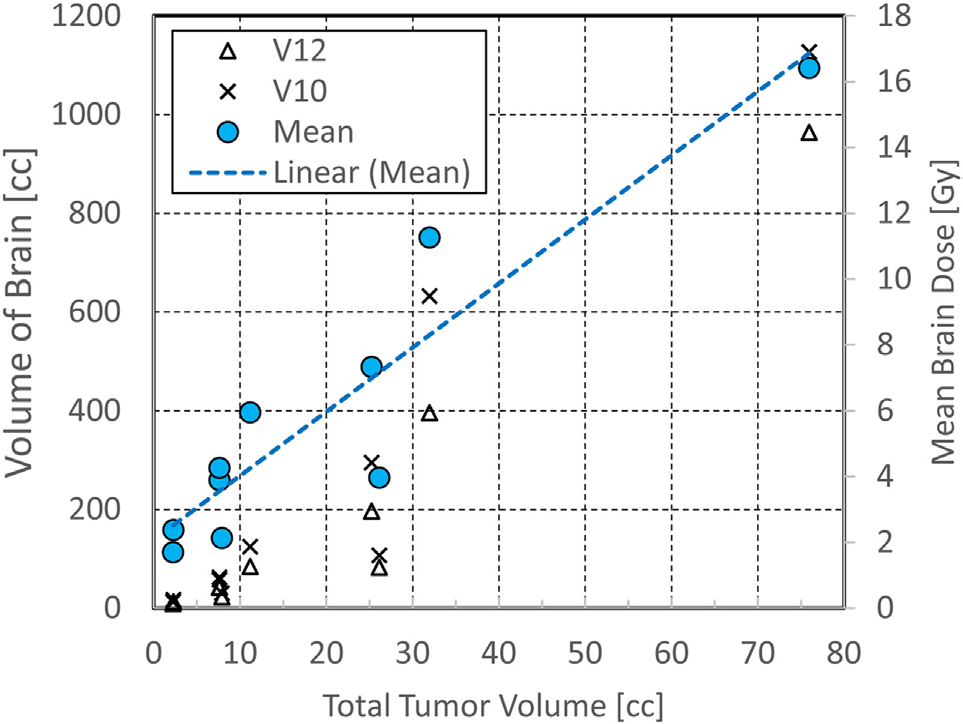
Correlation of the total tumor volume with the mean, V10 and V12 of whole brain. Correlation coefficient was calculated for the mean brain dose.

**Figure 4:**
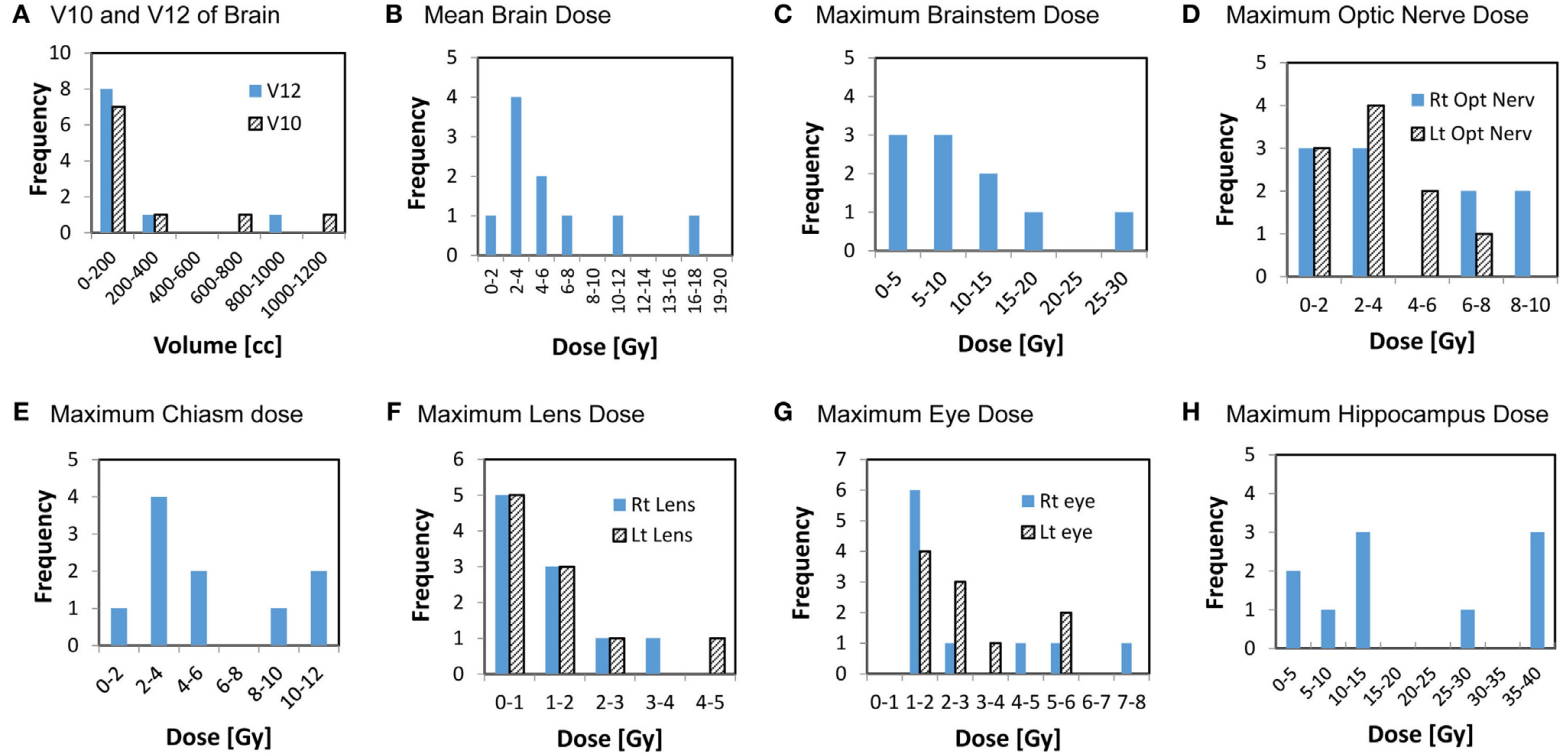
Histograms showing the distributions of the cumulative doses to critical structures from all GK sessions. Y axis represents the number of patients, x-axis represents dose or volume categories.

The cumulative doses to critical normal structures from all GK sessions were calculated using Velocity™ software for each patient, and the results are presented in Table 3. The mean cumulative dose to the whole brain ranged from 1.7 to 16.4 Gy with a median of 4.1 Gy. The medians of the volume of the brain receiving 12 Gy or higher (V12) and 10 Gy or higher (V10) were 63.1 cc (range, 9.6–964 cc) and 84.5 cc (range, 12.9–1,127 cc), respectively. As expected, these doses appear to correlate with the total tumor volume for each patient (Figure 3). The mean brain dose increased linearly as the total tumor volume increased, with a correlation coefficient of 0.932. The maximum doses to the chiasm, right optic nerve, and left optic nerve varied among patients with a median of 3.9 Gy (range, 1.8–10.8 Gy), 2.9 Gy (range, 1.2–9.0 Gy), and 2.6 Gy (range, 1.0–6.5 Gy), respectively. With regard to the brainstem, the median of the maximum cumulative doses was 6.1 Gy (range, 2.2–28.9 Gy). As expected, the medians of the maximum doses to the right and left lenses and the right and left eyes were low at 1.1, 1.1, 1.8, and 2.1 Gy, respectively. Finally, the medians of the mean and maximum cumulative dose to the hippocampi were 3.4 Gy (range 1.0–14.4) and 13.8 Gy (range, 1.5–39.3 Gy), respectively. It is noted that one patient (ID = 8) was treated for 63 metastases to a total tumor volume of 75.9 cc over seven sessions, spanning 427 days. Not surprisingly, this patient had the highest maximum cumulative doses to the brainstem (28.9 Gy) and hippocampi (39.3 Gy) among the entire cohort. The distribution of the cumulative doses to each structure was also plotted as histograms for all 10 patients (Figure 4).

**Table 3:** Cumulative doses to critical normal structures.

Dose (Gy) or volume (cc)													
	Brain	Brain DVH		Brainstem	Chiasm	Rt opt nrv	Lt opt nrv	Rt lens	Lt lens	Rt eye	Lt eye	Hippocampi	
Pt ID	Mean	V12 (cc)	V10 (cc)	Max	Max	Max	Max	Max	Max	Max	Max	Max	Mean
1	5.95	84.38	124.93	6.46	4.89	8.87	2.56	0.41	0.16	2.44	2.16	29.01	3.26
2	11.27	395.92	632.58	10.70	8.78	6.39	5.34	3.92	4.15	7.35	5.51	11.24	7.72
3	2.38	13.03	17.6	2.82	2.75	2.91	2.63	1.31	1.29	1.96	2.03	38.15	2.84
4	2.14	22.93	30.49	17.46	3.74	1.33	1.75	0.82	0.96	1.14	1.41	35.14	7.61
5	3.9	42.97	62.18	5.18	3.69	2.56	2.55	1.65	1.61	4.41	5.18	14.58	3.55
6	1.7	9.63	12.87	2.16	1.75	1.22	1.02	0.17	0.45	1.05	1.37	1.50	0.95
7	7.33	197.06	295.43	5.72	2.79	1.50	1.08	0.85	0.78	1.33	1.03	7.18	1.94
8	16.42	964.19	1,127.46	28.89	10.79	8.97	6.50	2.99	2.23	5.19	3.73	39.31	14.35
9	3.97	83.21	106.8	0.66	10.70	7.73	4.63	0.82	0.96	1.14	1.41	13.04	6.78
10	4.27	41.69	57.44	13.70	4.08	2.88	3.47	1.39	1.80	1.60	2.86	4.67	3.11
Mean	5.93	185.50	246.78	9.38	5.40	4.44	3.15	1.43	1.44	2.76	2.67	19.38	5.21
SD	4.67	298.06	362.89	8.67	3.39	3.19	1.83	1.17	1.14	2.16	1.62	14.55	3.99
Median	4.12	63.09	84.49	6.09	3.91	2.90	2.60	1.08	1.13	1.78	2.10	13.81	3.41

*V12, the volume receiving 12 Gy or higher; V10, the volume receiving 10 Gy or higher; Rt, right; Lt, left; opt nrv, optic nerve*.

**Figure 5:**
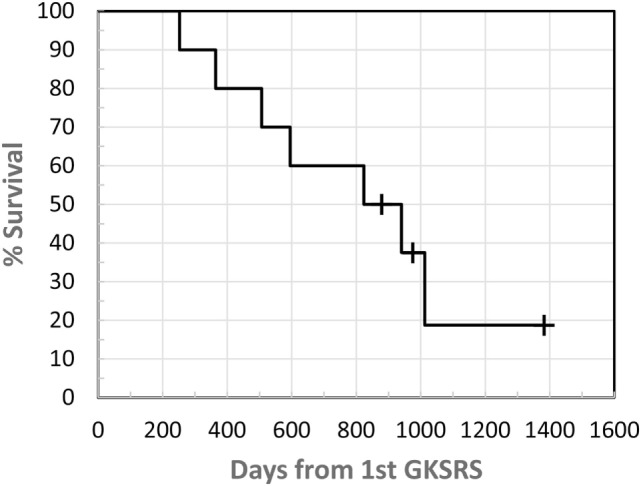
Kaplan–Meier survival plot. Survival was measured from the time of first GKSRS.

### Clinical Outcome

With a median follow-up of 852 days, 7 patients succumbed to their disease between 252 and 1,012 days following their first GKSRS (Figure 2). Three patients are still alive with their last clinic follow-up between 879 and 1,382 days. The median survival was about 800 days (~26.7 months or 2.2 years) for all 10 patients. None of the GK treated lesions developed radiation necrosis on follow up MRIs. No clinically significant treatment-related toxicity was noted during the follow-up period.

## Discussion

In this retrospective analysis, we reported the dosimetric and clinical outcome of 10 patients who received multisession GKSRS for recurrent BM and demonstrated the feasibility of such an approach. Our study was prompted by the lack of data concerning the treatment of multiply recurrent BM using repeat courses of SRS. While SRS has emerged as a preferred treatment modality for patients who present with a limited number of BM, there has not been a consensus regarding the optimal treatment strategy for patients who recur following initial SRS. According to a recent survey conducted by Sandler et al. for single course SRS treatments, there was a significant difference in the maximum number of lesions that should be treated with SRS versus WBRT among various radiation oncologists (17). High-volume practitioners specializing in CNS tended to have a higher cutoff number for SRS than all other radiation oncologists surveyed.

There are only a handful of studies examining repeat courses of SRS (18–23). Hillard et al. identified 10 patients who underwent at least 2 SRS treatments to 3 or more tumors (19). With a mean of 2.4 treatment sessions and 5.7 total tumors treated per patient, they determined that the maximum dose to critical structures remained remarkably small, ranging from a mean of 1.59 Gy to the left optic nerve and 10.59 Gy to the brainstem. The authors suggested that the impact of dose received in previous SRS treatments may have negligible toxicity for future SRS treatments. In another study by Shultz et al. (22), the authors identified 652 metastases treated in 95 patients over 273 courses of SRS. With a median of two courses (range 2–14) of SRS per patient and a median of two metastases (range, 1–14) per course of SRS, they found that this approach was relatively safe and effective, with adverse radiation events occurring in only 2% of SRS sites. The patients who developed clinically significant toxicity were those who received treatment to resection cavities, likely due to the larger volume of resection cavities compared to intact tumors. Furthermore, the authors showed that the aggregate tumor volume over multiple courses of SRS, rather than the cumulative number of metastases, was a predictor of overall survival. A recent study by Kotecha et al. focused on the intracranial recurrence pattern and salvage therapies for 59 patients who underwent ≥3 courses of SRS to a total of 765 different brain lesions (23). As expected, distant intracranial recurrence was frequent (64% at 6 months). However, QOL was preserved or improved in the majority of patients. Moreover, radiation necrosis occurred in only 10 patients (17%). The authors concluded that consideration should be given to additional courses of SRS for select patients with favorable risk factors upon intracranial relapse. Similarly, our patients were treated with 3–7 courses of GKSRS with a median of 5 courses. The total number of metastases treated ranged from 10 to 63 (median = 25). Taken together, these studies support the selective use of repeat SRS for a unique group of patients in the setting of multiply recurrent BM.

One of the concerns of using a repeat SRS approach is the potential toxicity to the normal structures from treatment to a large number of lesions over time. Few studies performed detailed dosimetric analyses to correlate with clinical outcomes. Using Velocity™ to calculate cumulative doses, we found the doses to various critical normal structures to be generally quite low. For example, the median doses to the lens and the eyes were in the range of 1.0–2.0 Gy, and the median of the maximum doses to the right and left optic nerves were 2.9 and 2.6 Gy, respectively, with the highest dose being 9.0 Gy in a single patient. Additionally, the median of the mean dose to the brain was only 4.1 Gy. Several studies examined the value of the V10 and V12 in predicting radio-necrosis following SRS and found an increased risk when V12 exceeds approximately 10 cc, and V10 exceeds approximately 12 cc (24). In our study, the median V12 was 63.1 cc, and the median for V10 was 84.5 cc, both significantly higher than the typical constraints used for SRS. However, one crucial difference is that our patients were treated over multiple sessions spaced by months of treatment-free intervals. In particular, for the two patients who received seven sessions, total treatment span was 427 days and 1,197 days, respectively. Presumably, repair may have taken place between treatments. The fact that none of our patients experienced radio-necrosis is in keeping with previously reported low necrosis rate and the suggestion that the impact of dose received in previous SRS treatments may be negligible on toxicity from future SRS treatments (19, 22). This may explain the lack of seeming toxicity in the patient with a cumulative maximum brainstem dose of 28.9 Gy.

A significant advantage of SRS over WBRT is its ability to preserve neurocognitive function. When many lesions are treated over multiple sessions, however, the cumulative dose to the hippocampus could potentially negate the benefit from SRS. The median of the mean cumulative dose to the hippocampus among our patients was 3.4 Gy, well below the dose constraint used for the RTOG 0933 of 10 Gy to 100% of this critical structure. Although a direct comparison is not possible without invoking a complex and validated radiobiological model comparing single vs. fractionated regimens, the cumulative doses found in our study compare favorably to that in the hippocampal sparing clinical trial, which demonstrated improved memory. Even in the patient who received GKSRS to a total of 63 lesions in 7 courses, the cumulative dose to the hippocampus was only 14.4 Gy. Some of the lesions were in the vicinity of the hippocampus, but the sharp dose falloff from SRS still resulted in a relatively low overall dose to this critical structure.

Although 7 of the 10 patients ultimately died of their disease, the survival of our cohorts compares favorably to the median survival of 10.8 months reported by Yamamoto et al. among patients treated for five to ten tumors (11) and the median survival of 6.9 months reported by Sperduto et al. in patients with a GPA of 3 (25). The median follow-up of our patients was 852 days, and the median survival was approximately 800 days (26.7 months). The prolonged survival observed in our patients strongly argues for strategies to identify the subset of patients with a good prognosis and for whom targeted therapy or immunotherapy is available. These patients would potentially benefit from repeat courses of SRS as opposed to conventional WBRT when new BM develops. The detrimental effects of treatment on QOL become an important consideration as survival times improve (18–21, 23, 26).

There are several limitations to our study. First, our patient size is small with only 10 patients. This is expected given our intention to analyze only those patients who were treated with three or more SRS sessions to ten or more lesions. Second, selection bias also played a strong role in the decision to treat new BM with repeat SRS. The patients selected for multisession SRS generally exhibited a good performance status and had systemic therapy options available. The patients’ prolonged survival made it possible to offer treatment strategies consisting of repeat GKSRS courses. Patients with poorer performance statuses and shorter life expectancies were more likely treated with WBRT rather than SRS and were, therefore, not included in this study. Third, these patients were treated with Leksell Gamma Knife 4 C, which is not the most advanced Gamma Knife system. With newer models such as Perfexion and Icon, better target conformity and steeper dose gradient can be achieved (27, 28). Consequently, we would expect even lower cumulative doses to critical organs if these patients were treated with a more modern treatment device. Fourth, the Velocity™ software we used to quantify the total cumulative dose to various structures does not contain an algorithm with a validated linear-quadratic (LQ) model to allow for comparison with a fractionated treatment scheme. We were also unable to account for the repair that may have occurred between the treatment sessions using Velocity™. Furthermore, the biological effect to a critical structure is determined not only by the cumulative dose from all treatment sessions but also by the differential contributions from each GK exposure, with the latter likely a more critical player. Thus, a new dose summation algorithm incorporating the LQ model as well as taking into account complex spatial and temporal factors will be required to more accurately quantify the true radiobiological consequences of SRS treatments interspersed with varying time-spans between the sessions. Such a process is only possible through multi-institutional collaborative efforts. Finally, none of our patients had formal neurocognitive testing and/or QOL assessment. Therefore, even though the cumulative doses to the hippocampus and various other organs appear to be low in our cohort, the clinical benefit of multisession SRS still needs to be validated.

Despite the feasibility demonstrated in our study, we caution that repeat GKSRS should only be entertained after careful evaluation of several important factors. We recommend that only patients with good performance status, well-controlled systemic disease, and a limited number of lesions be considered. Additionally, a longer treatment interval between sessions is preferred to capitalize the damage repair process. When new lesions appear in close proximity to the critical structures and/or cumulative tumor volume is estimated to be high, it is imperative to perform a pre-plan and dose summation and be prepared to resort to fractionated therapy if the dose to critical structures is prohibitive.

## Conclusion

Multisession SRS was safely delivered to patients with good performance status, well-controlled systemic disease, and a limited number of multiply recurrent BM. Despite re-treatment, a low dose to critical normal structures could be maintained. The low cumulative doses to the brain and the hippocampi may potentially spare these patients from radiation-induced neurocognitive decline as commonly seen with WBRT. This is becoming more critical with improved systemic therapy and longer survival in select patients. While the results of our study show potential promise of improving patients’ QOL *via* low doses to critical normal tissues, this approach does not represent the standard of care. Further studies will be required to evaluate the safety and benefit of multisession SRS fully.

## Ethics Statement

The study was approved by University of Minnesota IRB Code Number 0801M23942.

## Author Contributions

Conception and design: YW. Data collection: RL, KD, CL, DM, PS, JY, and YW. Data analysis and interpretation: RL, JY, and YW. Manuscript writing: RL, JY, DM, and YW. Final approval of manuscript: JY and YW.

## Conflict of Interest Statement

The authors report no conflict of interest concerning the materials or methods used in this study or the findings specified in this paper.

## Abbreviations

SRS, stereotactic radiosurgery; GK, gamma knife; RTOG, radiation therapy oncology group; WBRT, whole brain radiation therapy; BM, brain metastasis.
